# The Impact of Caregiving Burden on Mental Well-Being in Coronary Artery Bypass Graft Surgery Caregivers: The Mediatory Role of Perceived Social Support

**DOI:** 10.3390/ijerph18105447

**Published:** 2021-05-19

**Authors:** Claudio Singh Solorzano, Elizabeth Leigh, Andrew Steptoe, Amy Ronaldson, Tara Kidd, Marjan Jahangiri, Lydia Poole

**Affiliations:** 1 Department of Psychology, Sapienza University, Via dei Marsi 78, 00185 Rome, Italy; claudio.singh@uniroma1.it; 2 Department of Epidemiology and Public Health, University College London, 1-19 Torrington Place, London WC1E 6BT, UK; e.leigh@ucl.ac.uk (E.L.); a.steptoe@ucl.ac.uk (A.S.); 3 Department of Psychological Medicine, Institute of Psychiatry, Psychology and Neuroscience, King’s College London, London WC2R 2LS, UK; amy.ronaldson@kcl.ac.uk; 4 Department of Psychology, Faculty of Health, Liverpool John Moores University, Tom Reilly Building, Byrom Street, Liverpool L3 3AF, UK; T.M.Kidd@ljmu.ac.uk; 5 Department of Cardiac Surgery, St George’s Hospital, University of London, Blackshaw Road, London SW17 0QT, UK; marjan.jahangiri@stgeorges.nhs.uk; 6 Institute of Health Informatics, University College London, 222 Euston Road, London NW1 2DA, UK

**Keywords:** caregiver burden, social support, depression, well-being, coronary artery bypass graft surgery, caregiving

## Abstract

An increase in caregiver burden and a decrease in social support have both been identified as predictors of poor caregiver psychological distress. However, little is known about the role of these factors in coronary artery bypass graft (CABG) caregivers. The purpose of this study was to investigate whether change in perceived social support from pre to post surgery mediated the relationship between change in caregiver burden and caregiver depressive symptoms and subjective well-being post surgery. A sample of 101 caregivers of elective CABG patients were assessed 28 days before and 62 days after patients’ surgery. Caregivers completed the Oberst Burden Scale, the Enhancing Recovery in Coronary Heart Disease (ENRICHD) Social Support Instrument, the Beck Depression Inventory, and the Control, Autonomy, Self-Realisation, and Pleasure (CASP-19) scale. Simple mediation analyses showed that change in social support significantly mediated both the relationship between change in caregiver burden and post-surgery depressive symptoms (unstandardised β = 0.041, 95% CI (0.005, 0.112)) and the relationship between change in caregiver burden and post-surgery subjective well-being (unstandardised β = 0.071, 95% CI (0.001, 0.200)). Psychological interventions aimed at the CABG caregiver population should promote social support to deal with the increase of caregivers’ tasks and demands after the patients’ surgery.

## 1. Introduction

Recent estimates from the World Health Organisation (WHO) reveal that coronary heart disease (CHD) is the leading cause of death worldwide, accounting for more than 9 million global deaths in 2016 [1]. Coronary artery bypass graft (CABG) is one of the most common revascularisation procedures for advanced and extensive CHD [2]. In 2015, over 16,000 CABG procedures were performed in the UK [3], and over 159,000 in the USA in 2016 [4]. After the surgery, CABG patients are usually discharged from hospital within a week and can be expected to make a full physical recovery in two to three months depending on their fitness, age, and underlying disease severity [5,6].

Previous studies have identified the spouse or cohabiting partner of the patient as the primary source of support following CABG, who adopt the role of primary caregiver and play a key role in the patient’s physical and emotional well-being and health behaviours [7,8]. The literature has highlighted that the caregiving situation and its impact on health varies depending on the caregiver’s relationship with the care recipient [9]. For instance, adult–child caregiver relationships might lead the adult to experience caregiving as a burdensome duty that interferes with other responsibilities, and this might aggravate their distress [10,11]. Although spouses might find the experience of caring for their partners normative [9,12], many studies have shown the highest levels of burden in spouses than other groups [13,14]. Therefore, these spousal caregivers represent a group at particular risk of mental health problems.

Caregiving for a person who has undergone CABG differs from caregiving related to other chronic conditions, such as dementia or cancer. Whereas caring for a patient with a progressive illness is a long-term experience, which often ends with the patient’s death, a CABG caregiver might expect to begin their caregiving role at the point of the surgery and for it to end two to three months later [7]. Therefore, caregiving for a CABG patient provides an interesting model to investigate the experience of transient caregiving and the effects of a newly adopted role. Moreover, a focus on the first months after the surgery allows researchers to investigate the experience of CABG caregivers in the patient’s acute recovery period, and the associated mental health outcomes.

CABG caregivers report significant burden during the first year following the patient’s surgery [8,15]. Halm and colleagues [7] showed that concerns about their preparedness and the lack of information about patients’ needs after discharge were the main causes of stress and burden during the first three months of caregiving. Greater depressive symptoms [16,17] and poorer mental health [18,19] are common negative outcomes for CABG patient caregivers, but less is known about the effect of caregiving on positive aspects of affect.

Subjective well-being consists of hedonic and eudemonic aspects of mental health that are distinct from depressive symptoms [20]. Hedonic well-being refers to the experience of a positive emotion such as pleasure or happiness, while eudemonic well-being refers to the satisfaction of basic psychological needs and self-realisation [21]. Greater subjective well-being is generally considered to be health protective across a variety of health conditions [22]. To date, the only study that has analysed some aspects of well-being in CABG caregivers found that the most burdened caregivers also had the most personal gain and competence; conversely, they also had lower mental health-related quality of life [18]. These results present a paradox, suggesting the need for further research to understand the relationship between caregiver burden and subjective well-being over time.

The Stress Process Model (SPM) [23] proposes that the relationship between primary stressors (e.g., caregiver burden) and negative outcomes (e.g., depression, lower well-being) can be mediated by personal and social resources such as resilience, coping strategies, and social support. A robust body of knowledge has revealed both the health-negative effects of lack of social support, as well as the health-protective effects in a variety of caregiver domains, including both dementia and cancer [24,25]. A recent review indicated that caregivers of people with heart failure, chronic obstructive pulmonary disease, or coronary artery disease reported negative social support experiences after the patient’s hospital discharge, such as lack of family support and feeling of abandonment by healthcare teams [26]. In CABG caregivers, Kings and colleagues [27] reported a significant decrease of perceived social support from pre to post surgery, but there is a lack of more recent studies assessing the experience of social support in CABG caregivers. Moreover, only a few studies have investigated the effects of social support on well-being in CABG caregivers [27,28,29]. For instance, lower social support predicted disrupted mood [27] and poorer health-related quality of life [30]. Thomson and colleagues’ [28] results suggested a dyadic relationship between patient and caregiver well-being, such that the patient’s support was associated with the partner’s mental health status. However, to date, no studies have explored the possible effect of social support on the relationship between CABG caregiver burden and psychological well-being.

The aim of the present study was to examine the impact of caregiver burden over time on post-surgical depressive symptoms and subjective well-being in CABG caregivers. We also examined the role of change in perceived social support as a mediator of this relationship, to estimate the extent to which social support mediated the negative effects of caregiving burden on caregivers’ distress (i.e., depressive symptoms and subjective well-being).

## 2. Materials and Methods

### 2.1. Participants

**Figure 1:**
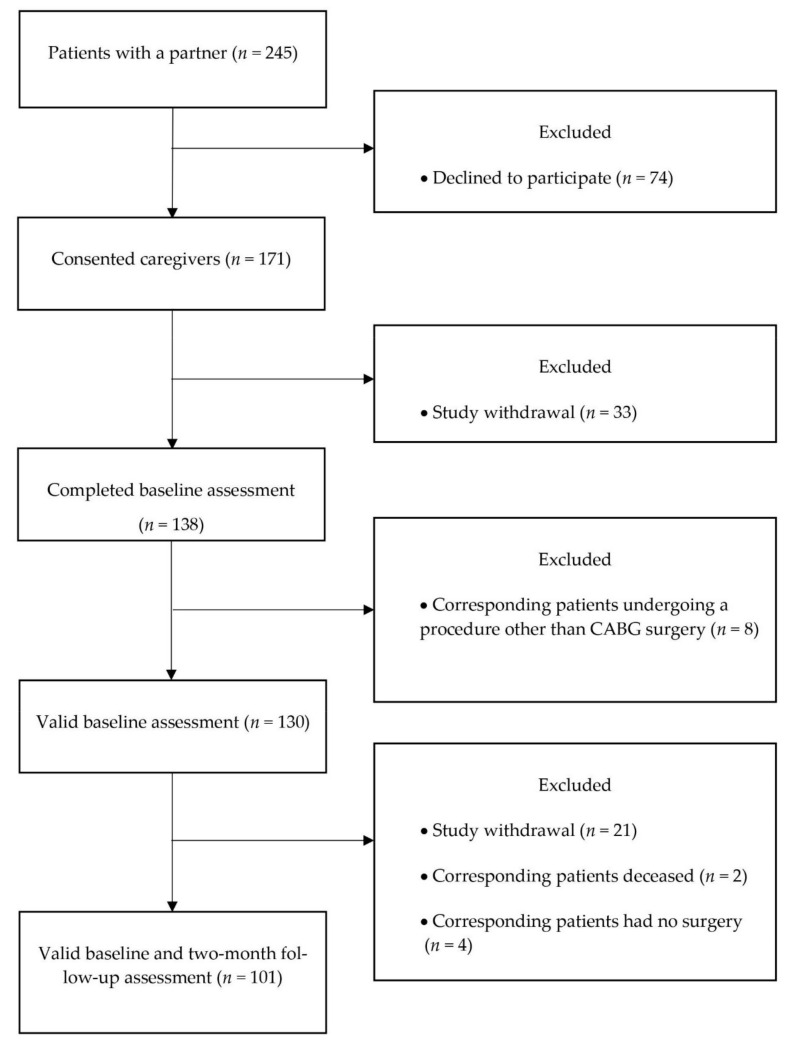
Flow diagram of caregiver participants’ progression through the ARCS (Adjustment and Recovery after Cardiac Surgery) study. Coronary artery bypass graft (CABG).

Participants were the caregivers (spouse or co-habiting partner) of patients who took part in the ARCS (Adjustment and Recovery after Cardiac Surgery) study. In brief, patients were recruited consecutively from a pre-surgery assessment clinic at St. George’s Hospital, London, between January 2010 and July 2012. Eligible participants had to undergo elective CABG surgery or CABG plus valve replacement, be at least 18 years of age, and had to be able to complete questionnaires in English. Full details are published elsewhere [31,32]. Caregiver participants were recruited alongside patients and consequently were excluded if their corresponding patients were excluded. Caregivers were included if they were over the age of 18, assuming the primary caregiving role, and excluded if they had communication or cognitive impairments and were unable to complete the questionnaires in English. The recruitment and retention of caregivers in the ARCS Study are displayed in Figure 1. Out of the 130 caregivers who completed valid baseline measurements, those participants included in these analyses were the 101 caregivers of CABG surgery patients with complete data for all variables at baseline and follow-up, including covariates. Compared to the caregivers who completed assessments at both time-points, those who did not complete the two-month follow-up were more likely to have high levels of burden (*t* = 2.369, *p* = 0.019) and to perceive lower social support (*t* = −2.499, *p* = 0.014) at the pre-surgery condition.

At the pre-surgery assessment, participants completed self-report questionnaires at home on average 27.9 days before the patients’ surgery and returned them by post. The follow-up assessment occurred on average 62.0 days after the surgery and caregivers followed the same procedure as pre surgery, completing self-report questionnaires and returning them by post. The follow-up questionnaire was administered approximately two months after the surgery when all patients would be expected to have been discharged from hospital and to be recovering at home. This allowed us to capture the caregiving experience during the patients’ acute recovery period.

The study was conducted in accordance with the 1964 Declaration of Helsinki and its later amendments. All procedures were carried out with the written informed consent of the participants. We obtained ethical approval from the South West London Research Ethics Committee (Protocol code 09/H0708/38 and date of approval 29 November 2010).

### 2.2. Measures

#### 2.2.1. Predictor Variables: Caregiver Burden Change

Caregiver burden was measured using the 15-item Oberst Caregiver Burden Scale (OBCS) [33], in which items load onto three factors: direct care tasks (e.g., personal care), instrumental care tasks (e.g., medical or nursing treatment), and interpersonal care tasks (emotional support, ‘being there’ for your partner). Using a 5-point Likert-type scale, caregivers were asked to report both the amount of time needed to perform each caregiving task (objective or ‘time’ burden scale) and the level of difficulty associated with these responsibilities (subjective or ‘difficulty’ burden scale). The questionnaire has been validated for use in cardiac surgery caregivers [15] and it has been used almost exclusively in caregivers of patients undergoing CABG surgery [8,15,18]. A total burden score was obtained by calculating the square root of the product of the two subscales. At pre-surgery, Cronbach’s alphas were 0.90 and 0.88 for the ‘difficulty’ and ‘time’ burden subscales, respectively. At post-surgery, Cronbach’s alpha was 0.94 for the ‘difficulty’ and 0.91 for the ‘time’ burden subscale. Both subscales and total scores ranged from 15 to 75, with higher scores indicating greater burden. To make use of the repeated measures available in ARCS, we calculated a change score (subtraction of pre-surgery total burden score from post-surgery total burden score) to describe the extent to which burden increased or decreased from pre-surgery to post-surgery follow-up. Positive scores indicated an increase in total caregiver burden over time.

#### 2.2.2. Mediator Variables: Social Support Change

Social support was measured using the 7-item ENRICHD Social Support Instrument (ESSI) [34]. The items relate to structural (i.e., partner), instrumental (tangible), and emotional (caring) support. Example items include: “Is there someone available to you to give you good advice about a problem?”; “Can you count on anyone to provide you with emotional support”. The response categories are on a five-point Likert scale ranging from 1 (None of the time) to 5 (All of the time). Item 7 (“Are you currently married or living with your partner?”) was removed in this study since all participants were cohabiting with their partner. Responses were summed to produce a total score, ranging from 6 to 30; higher scores indicate greater social support. The Cronbach’s alpha for this questionnaire was 0.85 at pre-surgery and 0.89 at post-surgery. ESSI has been used previously in studies of different types of caregivers [35,36,37] and it has been validated for use as a short measure to screen for social support [34]. To illustrate the impact of the significant change of social support on post-surgery outcomes, we calculated a change score by subtracting pre-surgical social support from post-surgical social support; higher scores indicate an increase in social support over time.

#### 2.2.3. Outcome Variables: Depressive Symptoms and Well-Being

The Beck Depression Inventory (BDI) [38] was used to measure depressive symptoms pre-surgery and 2 months after the surgery. It is a 21-item questionnaire which asks the respondent to reflect on how they have been feeling over the past two weeks. The BDI was scored by summing each chosen answer (on a scale of 0 to 3); higher scores indicate greater emotional disturbance, with a range of 0 to 63. For descriptive analyses, the cut-off score of ≥10 was adopted to indicate significantly elevated depression symptoms since it has established sensitivity and specificity for detecting caseness [38]. The BDI has a high reliability—both in terms of internal consistency and stability, in the assessment of non-psychiatric populations [39]. It has previously been administered in studies of caregivers of patients from a variety of patient groups, including those hospitalised with cardiovascular disease [40]. Pre-surgical Cronbach’s alpha for this questionnaire was 0.84, and at post-surgery it was 0.79. The continuous post-surgery depressive symptoms score was used as one of our outcome variables in analyses.

Subjective well-being was measured using the CASP-19 (Control, Autonomy, Self-Realisation, and Pleasure), a scale designed to measure overall well-being in older adults [41]. Respondents are asked how often each statement applies to them on a 4-point Likert scale (from 0 = never to 3 = often). Higher scores indicate poorer well-being (total score range, 0–57). The Cronbach’s alpha for CASP-19 in this study was 0.88 at pre-surgery and 0.90 at post-surgery. Post-surgery subjective well-being score was used as one of our outcome variables in analyses.

#### 2.2.4. Covariates

Covariates were all measured at the pre-surgery baseline. Information on participants’ age, gender, number of people in the household (including the participant), occupation, smoking, and the baseline distress variable were recorded. Occupation was classified according to the Office of National Statistics Standard Occupation Classification (SOC) 2010 index [42] into 9 groups ranging from high to low occupation; we used SOC as an indicator of socioeconomic status (SES). Smoking was measured as a binary variable (smoker/non-smoker). Light physical activity was assessed using the two items on walking from the International Physical Activity Questionnaire (IPAQ) [43]. These items ask the number of days a participant walked for at least 10 min at a time in the past week, and the average length of time spent walking on one of those days. The score was used as a continuous variable with higher scores indicating greater levels of activity. Patients’ clinical cardiac disease severity was determined using the European System for Cardiac Operative Risk Evaluation (EuroSCORE) [44]. Items were scored in accordance with the ‘logistic EuroSCORE’ method using a logistic regression equation to generate a percentage mortality risk estimate; higher resulting scores indicate greater risk of mortality.

#### 2.2.5. Statistical Analysis

Summary scores were created for all variables, and paired *t*-tests were used to explore the change in burden, social support, depressive symptoms, and well-being from pre-surgery to post-surgery follow-up (*t* and *p* values are presented). Associations between variables of the study were assessed using Pearson’s correlation coefficients and all results are reported as Pearson’s *r* and *p*-value. Hierarchical linear regression analyses were used to examine the influence of change in caregiver burden (i.e., the change from pre- to post-surgery) (Δcaregiver burden) and change in social support (i.e., the change from pre to post surgery) (Δsocial support) on both depressive symptoms and subjective well-being measured two months after the patients’ CABG surgery. In the hierarchical regression analyses, we first examined the direct effect of Δcaregiver burden on the dependent variables in Step 2. In Step 3, we estimated the total effect of the same relationship controlling for the potential mediator (i.e., Δsocial support).

To estimate the significance of the indirect effect of Δsocial support on the relationship between Δcaregiver burden and mental health in CABG caregivers, bootstrap mediation analysis through the SPSS PROCESS macro was applied [45]. The method included 5000 bootstrap samples for coefficient and indirect estimation, and 95% bias-corrected confidence intervals (CI) for the indirect effect. We performed two models; the independent variable in both was Δcaregiver burden and the dependent variables were depressive symptoms (model 1) and subjective well-being (model 2). The mediator variable was Δsocial support. As a secondary analysis, we tested the reverse mediation model, with Δcaregiver burden entered as a proposed mediator of the relationship between Δsocial support and the dependent variables. These control analyses were performed since the independent variable and mediator were measured at the same time, capturing change from pre- to post-surgical condition.

In all models, age, sex, SES, number of people living in the household, smoking, light physical activity, patients’ EuroSCORE, and the baseline of the distress dependent variable (i.e., either depressive symptoms or subjective well-being) were entered as covariates. Variance inflation factor (VIF) values and tolerance values were generated for all regression models to assess multicollinearity and the assumption was not violated (VIF <10 and tolerance >0.1). Results are presented as standardised beta coefficients. The significance level was set to *p* < 0.05, with precise *p*-value reported for all test results. For the indirect effects model, we reported the effect size and the 95% bias-corrected CI. All data analyses were conducted using IBM SPSS Statistic version 24 (SPSS Inc., IBM, Armonk, NY, USA)

## 3. Results

### 3.1. Descriptive Analyses

Table 1 presents the descriptive characteristics of the sample. Caregivers had an age range between 39 and 88 years, were predominantly female (96.0%), and the majority lived with their spouse only (87.1%). Furthermore, most caregivers were non-smokers (97.0%).

Patients had an average age of 68.63 ± 8.63 years (range: 44–90 years), were predominantly male (96.0%), and were overweight (body mass index >25 kg/m^2^, 77.2%). The most common comorbidities in patients were hypertension (75.3%) and diabetes (15.8%). The majority of patients had on-pump cardiopulmonary bypass surgery in isolation (74.3%). The average length of postoperative hospital stay was 7 days, with a range of 4 to 22 days.

As shown in Table 2, caregiver burden significantly increased from pre to post surgery (*t* = −7.81, *p* < 0.001), although on average scores remained low (26.48 ± 7.85) post-surgery, indicating overall low levels of burden in this group. Moreover, while overall, levels of social support were high after surgery (25.74 ± 5.03), there was a significant decrease over time in caregiver-perceived social support (*t* = 6.51, *p* < 0.001). Depressive symptoms and subjective mental well-being did not change significantly over time (*t* = 0.84, *p* = 0.402 and *t* = −0.88, *p* = 0.380 respectively). Depression scores ranged from 0 to 33, with 23.2% of caregivers above the cut-off score of 10 at the post-surgery follow-up.

### 3.2. Correlations between Change in Caregiver Burden, Change in Social Support, and Psychological Distress Measures

Table 3 shows the correlation between the study variables. A significant negative correlation was found between change in caregiver burden and change in perceived social support (*r* = −0.213, *p* = 0.033). Change in caregiver burden was significantly correlated with depressive symptoms (*r* = 0.258, *p* = 0.009) and subjective well-being (*r* = 0.299, *p* = 0.002). Change in social support was significantly correlated with depressive symptoms (*r* = −0.226, *p* = 0.023) and subjective well-being (*r* = −0.295, *p* = 0.003). A positive significant correlation was found between depressive symptoms and subjective well-being (*r* = 0.681, *p* < 0.001) (N.B. higher CASP-19 scores indicate poorer well-being).

### 3.3. Caregiver Burden and Social Support in Predicting Depressive Symptoms at 2-Month Follow-Up

Table 4 (Step 2) presents the results of the hierarchical linear regression model examining the direct and total effects in the relationship between Δcaregiver burden (i.e., the change from pre to post surgery) and depressive symptoms at the 2-month post-surgery follow-up, adjusting for covariates. Step 2 of the model shows the direct effect; a greater increase in caregiver burden was a significant predictor of higher depressive symptoms (β = 0.171, *p* = 0.016).

In Step 3 of Table 4, social support was added to the model, to estimate the total effect (i.e., the association between Δcaregiver burden and depressive symptoms, while taking into account Δsocial support). Results indicated that a greater reduction in social support was a significant predictor of greater depressive symptoms (β = −0.193, *p* = 0.005), but caregiver burden was no longer significant (β = 0.129, *p* = 0.062).

**Figure 2:**
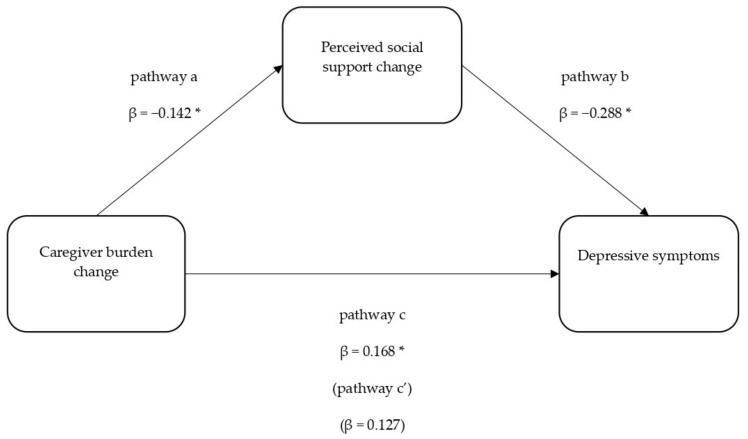
Mediation model of caregiver burden change and depressive symptoms through perceived social support change. * *p* < 0.05.

In the total effect model (i.e., Step 3 in Table 4), Δcaregiver burden was no longer significant, indicating the possible significance of the indirect effect in the relationship between Δcaregiver burden and depressive symptoms. Preacher and Hayes [45] bootstrapping estimates of indirect effects were employed. The overall model was significant (F(10,90) = 15.816, *p* < 0.001, Adj. R^2^ = 0.637). Figure 2 displays the unstandardised regression coefficients among the model variables. The total effect (pathway c) and the direct effect (pathway c’) pathways corroborate the results recorded in Table 4. The indirect effect (pathway ab) of Δsocial support in the model was significant (unstandardised β = 0.041, SE = 0.025, 95% CI (0.005, 0.112)), indicating that reduction in perceived social support over time significantly and fully mediated the relationship between greater caregiver burden over time and depressive symptoms.

### 3.4. Caregiver Burden and Social Support in Predicting Subjective Well-Being at 2-Month Follow-Up

Table 5 (Step 2) presents the results of the hierarchical regression model examining the direct and total effects in the relationship between Δcaregiver burden and subjective well-being at 2-month post-surgery follow-up, adjusting for covariates. The direct effect analyses showed that greater caregiver burden over time was significantly associated with lower post-surgical well-being (β = 0.136, *p* = 0.034).

In the total effect model (Step 3 of Table 5), a greater reduction in social support (i.e., the change from pre- to post-surgery) was significantly associated with lower post-surgery subjective well-being (β = −0.233, *p* < 0.001), whereas Δcaregiver burden was no longer significant (β = 0.086, *p* = 0.153).

**Figure 3:**
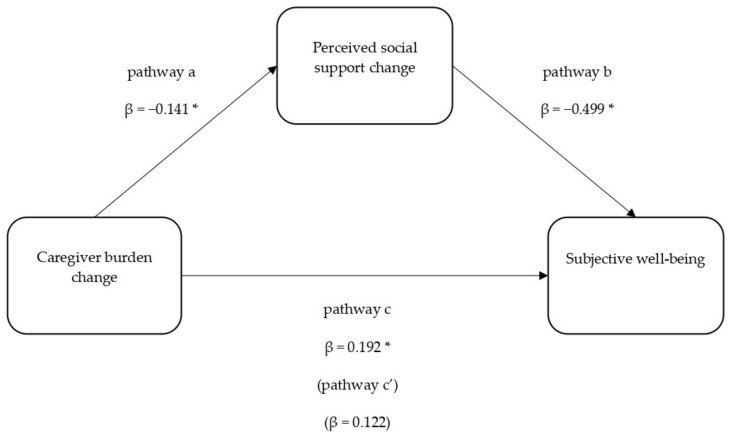
Mediation model of caregiver burden change and quality of life through perceived social support change. * *p* < 0.05.

Since Δcaregiver burden was no longer significant in the direct effect model, a mediation analysis was conducted to examine the possible indirect effect in the relationship between Δcaregiver burden and subjective well-being. The overall model was significant, (F(10,90) = 24.047, *p* < 0.001, Adj. R^2^ = 0.728). Figure 3 displays the unstandardised regression coefficients among the model variables. The results of the total effect (pathway c) and the direct effect (c’) of caregiver burden on depressive symptoms corroborated the results presented in Table 4. The indirect effect (ab) of social support in the model was significant (unstandardised β = 0.071, SE = 0.051, 95% CI (0.001, 0.200)), indicating that reduction in perceived social support over time significantly and fully mediated the relationship between greater caregiver burden over time and subjective well-being.

### 3.5. Secondary Analyses: Reverse Mediation

Since our predictor variable and mediator captured change over the same time period (pre-surgery to post-surgery), we explored the directional specificity of significant effects by running reverse mediation models. The first model examined the mediation role of Δcaregiver burden on the relationship between Δsocial support and depressive symptoms at the 2-month follow-up. The results showed that Δcaregiver burden did not mediate the relationship between social support and depressive symptoms (indirect effect (ab): β = −0.041, 95% CI (−0.152, 0.015)).

The second model investigated whether Δcaregiver burden meditated the relationship between Δsocial support and post-surgical subjective well-being. ΔCaregiver burden did not show a significant indirect effect in this relationship (indirect effect (ab): β = −0.038, 95% CI (−0.189, 0.012)). These findings support our previous models in which Δsocial support mediates the relationship between Δcaregiver burden and distress (i.e., depressive symptoms and subjective well-being) in CABG caregivers as opposed to the reverse.

## 4. Discussion

The present study investigated the influence of caregiver burden on post-surgical depressive symptoms and subjective well-being in CABG caregivers. Moreover, the mediation effect of change in social support in this relationship was examined. The results indicated that while an increase in caregiver burden over time was associated with increased depressive symptoms and lower subjective well-being, a decrease in social support over the same time mediated these relationships. These findings are the first to our knowledge to assess the indirect effect of reduced social support on the relationship between caregiver burden and depressive symptoms and subjective well-being in CABG caregivers.

In the present study, we found that caregiver burden was significantly associated with depressive symptoms. In particular, caregivers with greater increase in burden following the patient’s surgery showed the highest depressive symptoms at the 2-month post-surgery follow-up. In prior studies on CABG caregivers, post-surgical depressive symptoms ranged from 9% [46] to 67% [16], with the disparity in findings across studies partly accounted for by the sample (e.g., the inclusion of myocardial infarction caregivers). We found that almost one-quarter (23.2%) of CABG caregivers had elevated depressive symptoms, confirming the importance of this adverse psychological outcome in this sample and the need for appropriate interventions. Some prior studies have explored the cross-sectional relationship between CABG caregivers’ burden and depressive symptoms pre-surgery [47] and post-surgery [48,49], but no studies have looked at these relationships over time. Mediation analyses indicated that the relationship between change in caregiver burden and depressive symptoms was explained by change in social support. This finding is in line with SPM [23] in which a stressful condition (i.e., caregiver burden) might lead to the development of undesirable psychological outcomes (i.e., depressive symptoms) through a mediator, such as a decrease in perceived social support [23]. Other studies have also shown a mediating effect of social support on the pathway from stress to depression in other caregiving populations [50,51]. Our results have extended these findings to CABG caregivers for the first time.

We also found that change in caregiver burden showed a significant association with subjective well-being. In particular, caregivers with a greater increase in burden during the patient recovery period showed poorer subjective well-being at the 2-month post-surgery follow-up. This result is inconsistent with the only study investigating caregiver well-being in which higher burden scores were associated with increased personal gain and caregiver competence [18]. However, in our study, we focused on the burden caused by entering into a caregiving role (i.e., change of caregiver burden from pre-surgery to post-surgery). In contrast, Halm and colleagues only investigated associations between post-surgery burden and well-being levels. In our study, further analyses indicated that change in social support was a significant mediator of the relationship between change in caregiver burden and subjective well-being. This result is again in line with theoretical frameworks such as SPM, as well as being congruent with studies analysing the pathway between burden and subjective well-being in other caregiving populations [52,53].

We found relatively low post-surgery levels of caregiver burden and high levels of social support despite the significant detrimental change of these variables from the pre-surgery condition. However, the adverse psychological effects of higher caregiver burden and lower social support in other caregiver populations are well-known. For instance, the burden of caring for older adults [54,55], people with dementia [56,57], and cancer patients [58] has been shown to lead to lower mental health and subjective well-being. Moreover, the decline of social support has been associated with lower levels of psychological well-being in different types of chronic caregivers [59,60]. Our findings show that the effect of burden and social support on caregiver distress could also be extended to transient and temporary caregiving conditions, such as caring for a CABG patient.

Our findings shed new light on the pathways that link caregiver burden to psychological distress (i.e., depressive symptoms and subjective well-being) in CABG caregivers. While our findings are unable to make causal assertions due to the overlapping timeframe of our predictor and mediator variables, the reverse mediation models (i.e., change in caregiver burden mediating the relationship between change in social support and depressive symptoms and the relationship between change in social support and subjective well-being) were not significant, lending further support to the directional specificity of our results.

In our sample, caregiver burden significantly increased and social support significantly decreased from pre-surgery to post-surgery. The use of change scores in our analysis allowed us to clarify the impact of the intense and sudden role change of CABG caregivers from pre-surgery to post-surgery reported in other studies [26]. One possible explanation for this relationship between caregiver burden and social support is related to our caregiving burden measure that combined aspects of time and difficulty. Therefore, the increase in time needed to perform caregiving tasks and the difficulty associated with these responsibilities might negatively impact an individual’s capacity to commit to sustaining their social network, thus reducing the support received from them [55,61]. A further potential explanation for this finding is that the perception of social support could be affected by the new and stressful role of caregiving. Many studies have confirmed the difference between received and perceived social support and their different effects on mental and physical well-being [62,63]. The perception of social support is based for the most part on personal evaluative processes and characteristics [62,64]. It is possible that the new context of CABG caregiving and the change in the relationship with the patient might affect caregivers’ needs and characteristics [7,46] leading to a change in how they perceive the supportive behaviours from their network [62,63,65]. The lack of perceived social support, in turn, might lead to increased depressive symptoms and poorer psychological well-being [66].

The importance of improving perceived social support has been demonstrated in intervention studies for caregivers that target different social support components [67,68]. There is a lack of research investigating the effects of social support interventions on CABG caregivers thought to be in part due to social and economic barriers (e.g., healthcare structure, lack of funding), as well as individual barriers (e.g., patient preferences for care) [69]. Hartford and colleagues [70] showed that an information and support telephone intervention after the patient’s discharge reduced CABG caregiver anxiety. In the context of the VITAL programme, a telephone support intervention on managing patient recovery as well as the practical needs and concerns of caregivers produced a greater reduction in CABG caregiver anxiety and depressive symptoms [71]. A recent review on social support interventions in dementia caregivers showed that internet-based and video-phone interventions might benefit caregivers’ perceptions of support and mental well-being [67]. Remote interventions have the advantage of being relatively low-cost, bringing the intervention into the caregiver’s home, and reducing problems of accessibility and time [72]. For instance, Dam and colleagues [73] showed that an intervention in which caregivers had to create an online support group with their family and friends, with which to manage caring activities, helped caregivers gain mutual support and improved care organisation. These studies indicate the potential benefits of multicomponent social support interventions on reducing the risk of adverse psychological outcomes in CABG caregivers. However, further research is needed to examine the long-term effects of such trials.

This study has several strengths. The longitudinal design allows us to explore the impact of the patient’s surgery on the caregivers’ psychosocial well-being over time. The change scores of burden and social support help achieve this aim, showing how entering a new caregiving role may alter the caregivers’ psychosocial experience. However, there are limitations in the present study that must be acknowledged. First, as previously mentioned, because change in caregiver burden and change in social support were measured at the same time, we are unable to establish causality. However, the null findings on the competitive indirect pathway model, along with the theoretical underpinnings (i.e., SPM) on which we base the present study, allow us to support the direction of our findings. Second, subjective well-being was measured using a self-report questionnaire that does not consider other aspects of positive affect, such as self-esteem and optimism. Third, using a questionnaire to measure depressive symptoms restricts us from generalising our results to clinical samples. Fourth, our sample was composed mainly of female participants, so our results may not be readily generalisable to male caregivers. Prior literature has indicated that women have a higher prevalence of mood and affective disorders than men [74]. However, this prevalence is explained by the predominance of men in the UK CABG surgical population. Fifth, the caregivers included in the study are all spouses or partners of patients. Since many studies have reported the difference in caregiving experiences between spousal caregivers and other caregivers groups [9], our results are limited to the caregiving experience in romantic relationships. However, this prevalence is typical in other CABG caregiving studies [26].

## 5. Conclusions

In conclusion, we found that change in perceived social support fully mediated the relationship between change in caregiver burden and post-surgery depressive symptoms in CABG caregivers. Moreover, change in perceived social support also fully mediated the relationship between change in caregiver burden and post-surgery subjective well-being. The present findings have important implications for understanding how a CABG caregiver’s new role can lead to increased psychological distress. Based on the current findings, our data support the use of interventions on CABG caregivers, with particular emphasis on the provision of more tangible and emotional support to deal with the caregiving scenario.

## Figures and Tables

**Table 1:** Baseline characteristics of the sample (N = 101).

Characteristic	Mean ± SD or *n* (%)
*Baseline demographic variables*	
Age	65.89 ± 8.48
Sex—Female	97 (96.0)
Occupation classification	
High	41 (40.6)
Intermediate	49 (48.5)
Low	11 (10.9)
Household of two persons	88 (87.1)
*Baseline behavioural variables*	
Smoker	3 (3.0)
Physical activity—walking (hours per week)	4.26 ± 5.58
*Patients’ clinical risk*	
EuroSCORE (%)	4.28 ± 3.58

**Table 2:** Psychosocial, biological, and health variables of the sample (*n* = 101).

Characteristic	Pre-Surgery	2-Month Follow-Up	Change Score
	Mean ± SD	Mean ± SD	Mean ± SD
Caregiver Burden (OCBS)	21.45 ± 5.17	26.48 ± 7.85	5.02 ± 6.47 *
Social Support (ESSI)	28.51 ± 4.65	25.74 ± 5.03	−2.76 ± 4.27 *
Depressive Symptoms (BDI)	7.18 ± 5.96	6.80 ± 6.34	
Subjective Well-Being (CASP-19)	15.38 ± 7.77	15.89 ± 9.15	

* *p* < 0.001. OBCS: Oberst Caregiver Burden Scale; ESSI: ENRICHD Social Support Instrument; BDI: Beck Depression Inventory; CASP-19: Control, Autonomy, Self-Realisation, and Pleasure Scale.

**Table 3:** Correlations between variables of the present study (*n* = 101).

Variable	1. Change in Caregiver Burden	2. Change in Social Support	3. Depressive Symptoms	4. Subjective Well-Being
1. Change in caregiver burden	-			
2. Change in social support	−0.213 *	-		
3. Depressive symptoms	0.258 **	−0.226 *	-	
4. Subjective well-being	0.299 **	−0.295 **	0.691 **	-

* *p* < 0.05; ** *p* < 0.01.

**Table 4:** Hierarchical linear regression of depressive symptoms on caregiver burden and social support (N = 101).

Predictor Variable	B	S.E.	95% CI	β	*p*
*Step 1*					
Age	0.032	0.071	[−0.109; 0.173]	0.043	0.652
Gender	2.538	2.300	[−2.031; 7.106]	0.078	0.273
Occupation	0.144	0.238	[−0.328; 0.616]	0.043	0.546
Number in household	1.455	0.752	[−0.039; 2.949]	0.149	0.056
Smoking	5.491	2.620	[0.286; 10.695]	0.148	**0.039**
Physical activity	0.146	0.083	[−0.019; 0.311]	0.128	0.083
Patients’ EuroSCORE	0.034	0.142	[−0.247; 0.316]	0.019	0.809
Baseline depressive symptoms	0.759	0.074	[0.612; 0.906]	0.714	**<0.001**
*Step 2*					
Age	0.019	0.069	[−0.118; 0.156]	0.025	0.785
Gender	1.664	2.267	[−2.839; 6.168]	0.051	0.465
Occupation	0.102	0.232	[−0.359; 0.563]	0.030	0.661
Number in household	1.068	0.749	[−0.420; 2.556]	0.109	0.157
Smoking	5.127	2.555	[0.051; 10.203]	0.138	**0.048**
Physical activity	0.132	0.081	[−0.029; 0.293]	0.116	0.108
Patients’ EuroSCORE	0.074	0.139	[−0.202; 0.350]	0.042	0.594
Baseline depressive symptoms	0.750	0.072	[0.607; 0.893]	0.705	**<0.001**
ΔCaregiver burden	0.168	0.068	[0.032; 0.303]	0.171	**0.016**
*Step 3*					
Age	0.007	0.067	[−0.126; 0.139]	0.009	0.918
Gender	2.320	2.195	[−2.040; 6.681]	0.072	0.293
Occupation	0.118	0.224	[−0.326; 0.562]	0.035	0.599
Number in household	0.808	0.726	[−0.625; 2.261]	0.084	0.263
Smoking	3.546	2.522	[−1.464; 8.555]	0.095	0.163
Physical activity	0.136	0.078	[−0.019; 0.291]	0.120	0.085
Patients’ EuroSCORE	0.083	0.134	[−0.183; 0.348]	0.047	0.539
Baseline depressive symptoms	0.758	0.069	[0.620; 0.896]	0.712	**<0.001**
ΔCaregiver burden	0.127	0.067	[−0.006; 0.260]	0.129	0.062
ΔSocial support	−0.288	0.101	[−0.487; −0.088]	−0.193	**0.005**

Bold font indicates statistical significance (*p* < 0.05). Step 1: F(8,92) = 15.752, *p* < 0.001, *Adj.* R^2^ = 0.541. Step 2: F(9,91) = 15.447, *p* < 0.001, *Adj.* R^2^ = 0.565; R^2^_change_ = 0.026, *p* = 0.016. Step 3: F(10,90) = 15.816, *p* < 0.001, *Adj.* R^2^ = 0.597; R^2^_change_ = 0.033, *p* = 0.005.

**Table 5:** Hierarchical linear regression of subjective well-being on caregiver burden and social support (N = 101).

Predictor Variable	B	S.E.	95% CI	β	*p*
*Step 1*					
Age	0.058	0.090	[−0.122; 0.237]	0.054	0.524
Gender	4.455	2.970	[−1.443; 10.353]	0.095	0.137
Occupation	0.395	0.305	[−0.212; 1.002]	0.081	0.199
Number in household	2.725	0.974	[0.791; 4.659]	0.193	**0.006**
Smoking	8.717	3.389	[1.987; 15.447]	0.163	**0.012**
Physical Activity	0.152	0.107	[−0.060; 0.364]	0.093	0.157
Patients’ EuroSCORE	−0.022	0.186	[−0.390; 0.347]	−0.008	0.907
Baseline well-being	0.910	0.074	[0.763; 1.057]	0.774	**<0.001**
*Step 2*					
Age	0.044	0.089	[−0.133; 0.221]	0.041	0.622
Gender	3.405	2.953	[−2.460; 9.270]	0.073	0.252
Occupation	0.343	0.301	[−0.255; 0.940]	0.070	0.257
Number in household	2.308	0.974	[0.372; 4.243]	0.164	**0.020**
Smoking	8.226	3.331	[1.609; 14.842]	0.153	**0.015**
Physical activity	0.134	0.105	[−0.074; 0.343]	0.082	0.204
Patients’ EuroSCORE	0.016	0.183	[−0.347; 0.379]	0.006	0.929
Baseline well-being	0.891	0.073	[0.745; 1.036]	0.757	**<0.001**
ΔCaregiver burden	0.192	0.089	[0.015; 0.369]	0.136	**0.034**
*Step 3*					
Age	0.025	0.083	[−0.139; 0.189]	0.023	0.762
Gender	4.536	2.754	[−0.934; 10.006]	0.097	0.103
Occupation	0.374	0.279	[−0.181; 0.928]	0.077	0.184
Number in household	1.885	0.910	[0.077; 3.693]	0.134	**0.041**
Smoking	5.477	3.166	[−0.813; 11.767]	0.102	0.087
Physical activity	0.143	0.097	[−0.050; 0.336]	0.087	0.145
Patients’ EuroSCORE	0.029	0.170	[−0.308; 0.366]	0.011	0.864
Baseline well-being	0.892	0.068	[0.757; 1.027]	0.759	**<0.001**
ΔCaregiver burden	0.121	0.084	[−0.046; 0.289]	0.086	0.153
ΔSocial support	−0.499	0.126	[−0.748; −0.249]	−0.233	**<0.001**

Bold font indicates statistical significance (*p* < 0.05). Step 1: F(8,92) = 22.680, *p* < 0.001, *Adj*. R^2^ = 0.634. Step 2: F(9,91) = 21.479, *p* < 0.001, *Adj.* R^2^ = 0.648; R^2^_change_ = 0.016, *p* = 0.034. Step 3: F(10,90) = 24.047, *p* < 0.001, *Adj.* R^2^ = 0.697; R^2^_change_ = 0.048, *p* < 0.001.

## Data Availability

Data sharing is not applicable to this article.
